# Ovarian tissue vitrification: the use of a novel metal closed system for clinical grade cryopreservation

**DOI:** 10.5935/1518-0557.20140086

**Published:** 2014

**Authors:** Douglas Aquino, Lucas Danielli, Paula Rigon, Nivia Lothhammer, Nilo Frantz, Adriana Bos-Mikich

**Affiliations:** 1 Department of Morphological Sciences ICBS. Federal University of Rio Grande do Sul, Porto Alegre, RS, Brazil; 2 Nilo Frantz Human Reproduction Centre Aquino, Porto Alegre, RS, Brazil

**Keywords:** metal cryovial, vitrification, ovarian tissue

## Abstract

**Objective:**

To investigate whether a metal chamber is an appropriate system for vitrification of ovarian tissue under clinical grade.

**Methods:**

Experimental study, control versus treatment. Bovine ovarian cortices cut in 1x1x1 mm fragments were vitrified using ethylene glycol and dimethyl sulfoxide inside steel cryovials, whose bases were in touch with Liquid Nitrogen (LN2). Screw caps closed the cryovials before plunging into LN2. Primordial (n=356) and primary (n=327) follicles and the stroma were analyzed after histological preparation using light microscopy.

**Results:**

High rate of primordial (93%) and primary (80%) follicles presented normal morphology in the rewarmed fragments. There was not a significant difference between controls and primordial follicles morphology (P=0.1519). Significant difference was observed for the primary follicles (P=0.0097). Important to point out that stromal cells and collagen fibers presented a remarkable integrity, without major alterations in the cryopreserved tissues.

**Conclusions:**

The steel cryovial seems to be a safe means of vitrification under clinical grade conditions, with very fast cooling rates and no direct contact of the biological material with the liquid nitrogen (LN2). Ovarian reserve represented by primordial and primary follicles and stroma are very well preserved in this vitrification system.

## INTRODUCTION

Research on female fertility preservation has shown considerable progresses particularly on the field of ovarian tissue cryopreservation. This technology represents an interesting option for young women facing a cancer treatment, because it may not only preserve their fertility, but also restore their ovarian function after autograft.

Different protocols have been developed in the last years aiming to increase the efficacy of vitrification, particularly for oocyte and embryo cryopreservation. In addition, it has also been shown that vitrification is a more efficient cryopreservation method for the preservation of ovarian structure and function (Tokieda *et al*., 2002; Keros *et al*., 2009). One of the most important factors to take into account when performing vitrification is the fact that it requires extremely fast rates of cooling and precise timings of exposure to cryoprotectants.

With one exception (Migishima *et al*., 2003), vitrification methods for ovarian tissue described so far (Sugimoto *et al*., 2000; Tokieda *et al*., 2002; Migishima *et al*., 2003; Almodin *et al*. 2004; Chen *et al*., 2006; Keros *et al*., 2009; Isachenko *et al*., 2009; Sheikhi *et al*., 2011) refer to open systems that expose the biological material to the liquid nitrogen (LN2). It has already been reported (Bielanski *et al*., 2000) that LN2 may be a potential source of pathogens, which may pass to the gamet, embryo or tissue that contact it directly. We have recently reported (Bos-Mikich *et al*., 2012) a successful vitrification protocol for mouse ovaries, in which we made use of an aluminium foil caddy as a vitrification vessel.

The metal container touched the LN2 surface and the ovarian tissue immediately vitrifies. The aluminium foil vessel was then inserted into a plastic cryovial, which has previously been immersed in the LN2, and tightly closed.

Our results showed that there were no differences in the morphology of primordial follicles between vitrified/rewarmed and control ovarian tissues, even when the ovaries remained for a period of up to 4 h at room temperature or 38^o^C before vitrification.

In sequence to the previous study performed using the murine model, the present research aimed to test a metal cryovial made of stainless steel, in which bovine tissue was vitrified and stored, without any contact with the LN2.

The viability of the vitrified tissue was measured in terms of normal primordial and primary follicular morphology and stromal components features.

## MATERIAL AND METHODS

### Animals

Bovine ovaries were transported to the laboratory within 2h after slaughter in sterile glass vessel containing about 500ml of saline solution at room temperature (RT; ~23^o^C). The study was approved by the Ethics Committee of our Institution (Federal University of Rio Grande do Sul).

### Vitrification

Cortex slices were cut in 1x1x1 mm fragments (Ferreira *et al*., 2011) with scalpel and transferred to equilibrium solution (ES) of 7.5% ethylene glycol (EG) and DMSO followed by the vitrification solution (VS) of 15% EG and DMSO, both in HTF (Irvine) medium, for 25 and 15 min, respectively. Fragments were gently transferred from one solution to another with the help of fine paintbrushes to avoid damaging the tissue carrying the least amount of medium in each transfer.

The base and lid of the metal cryovial were placed in a tray containing LN2 to cool down. From the VS, ovarian fragments were taken using the paintbrushes and placed inside the base of the cryovial. The cryovial lid was tightly screwed on the top of the base with the help of a pair of frozen forceps and the system was immediately immersed in LN2 for storage. Three experimental replicates using ten ovaries from slaughterhouse were performed.

Each cryovial received 10-12 pieces of tissue from different ovaries. At each experimental replica 4-5 fresh fragments from different ovaries were fixed as controls.

The cryovial was made of stainless steel, 1ml volume and a height of approximately 1 inch with the lid on. Inside the screw top teflon tape was placed to seal the lid.

### Rewarming

The cryovials were removed from the LN2, left at RT for 30 sec for rewarming, before exposure to water bath at 37ºC for 1 min.

The cryovial was opened and its contents were gently removed from the bottom and transferred to the first warming solution containing 1M sucrose for 1 min, followed by the second solution containing 0.5M sucrose for 3 min and the last solution of 0.25M sucrose for 5 min. All tissue handling and manipulations were performed with the help of paintbrushes.

Last, the tissue fragments were left for 5 min in HTF-Hepes medium before being fixed in Bouin´s solution for histology. All solutions were at room temperature.

### Light microscopy

Ovarian morphology, particularly primordial and primary follicles and the stroma were analyzed by light microscopy (X 400 magnification).

Warmed and control ovarian fragments were left in Bouin´s solution for 45 min and dehydrated in a series of ethanol at RT.

Next, tissue fragments were embedded in paraffin wax. Serial sections of 5 µm thickness were prepared.

Every 15th section of tissue was mounted on glass slides and stained with hematoxylin and eosin. In case of doubt and to avoid double counting, each follicle was followed through neighbouring sections to be counted only once. The developmental stages of the follicle were classified by the number of layers and shape of granulosa cells surrounding the oocyte.

Briefly, primordial follicles were classified as those containing a single layer of flattened granulosa cells and primary follicles were those with a complete single layer of cuboidal granulosa cells (Gougeon, 1996).

Criteria for considering a primordial or primary follicle as damaged included: presence of vacuoli in the oocyte ooplasm, pyknotic germinal vesicle, oocyte detachment from the follicle basal membrane (clear spaces between oocyte and follicle basement membrane), incomplete follicular cells layer surrounding the oocyte.

Secundary and antral follicles were not counted on our analysis, but we did take notes on their morphological appearance. Slides were analyzed under blind conditions by one trained embryologist.

### Leak-testing of the sealed cryovial

To test whether the cryotube was LN2 leak proof we did a simple procedure: inside the cryotube we put a dry red powdered dye (Sudan), before tightly screwing the lid of the tube and immersing it into LN2. In case LN2 enters into the tube the dye would change color from reddish to purple-black in an aqueous solution.

### Statistical analysis

Differences between groups were analyzed using x2. A value of P<0.05 was considered statistically significant.

## RESULTS

**Figure 1:**
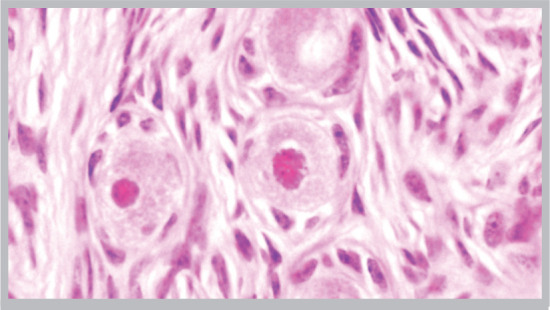
Light micrograph of vitrified-warmed bovine primordial follicles crypreserved in a metal cryovial (hematoxylin and eosin stained section of paraffin embedded tissue fragment; X 400). Note the well-preserved follicular cells and oocytes and the stroma cells and fibers.

In total, 683 fresh and cryopreserved primary and primordial follicles were analyzed. Our results show that 93% of the primordial and 80% of the primary follicles present in the rewarmed fragments showed normal morphology (Table 1; Figure 1).

**Table 1:** Numbers and percentages (%) of morphologically normal primordial and primary follicles, after vitrification in the steel cryovial and in fresh control ovarian fragments.

	Follicles	
	Primordial Normal/Total	Primary Normal/Total
Cryovial	216/232 (93%)	108/135 (80%)^a^
Controls	120/124 (97%)	173/192 (90%)^b^

Different subscripts within a column indicate significant differences among groups

These results show no significant difference in terms of normal morphology between rewarmed primordial follicles and fresh controls (P= 0.1519).

Primary follicles, however, presented a significant reduction in the number of morphologically normal structures after vitrification, when compared with controls (P=0.0097). The criteria used for classification as damaged or viable primordial and primary follicles was primarily based on the characteristics of the oocytes, as they are reported to be the first to die at these stages of development (Rajakoski, 1960; Rodgers & Irwing-Rodgers, 2010). In addition to the primordial and primary stage follicles, follicular and oocyte morphology was also analyzed in antral follicles, when the histological preparation presented a structure having more than one follicular cells layer and an antrum. We observed that five out of six of the growing antral follicles showed normal morphology after cryopreservation compared with four out of six and four out of four controls.

The normal morphology classification in these instances was based both on the oocyte normal characteristics (round, euchromatic nucleus and uniform cytoplasm without vacuoli or shrinkage) and round granulosa cells attached to the follicular wall and to the oocyte.

It was not possible to determine the incidence of apoptotic bodies in the membrane granulosa (Fair *et al*., 1997) of the growing antral follicles, as these structures are primarily observed in pre-ovulatory follicles, hardly detected in histological preparations. Further, mural and oocyte granulosa cells should isolated, fixed and specifically processed to assess apoptotic bodies, which is not possible in whole ovarian specimens. Stromal elements presented a remarkable recovery after cryopreservation. No alteration was observed in any of the tissue samples analyzed regarding the organization of the collagen fibers, the fibroblast nuclei and the wall organization of small blood vessels (Figure 1). We could state that stroma integrity after vitrification was as good as in control samples. Finally, the leak testing for the capacity of the cryotube to avoid LN2 to enter into it during storage showed a positive result, in the sense that the powdered dye remained dry and red inside the cryotube, after immersion and storage in L2i.

## DISCUSSION

Present results show a very good recovery rate of primary and primordial follicles, when viability is measured in terms of normal morphology, after vitrification of bovine ovarian cortical tissue using a metal container, without direct contact with LN2.

Primordial follicles survived at a rate of 93%, not statistically different from controls. This is a reassuring result considering that this system may potentially be employed in fertility preservation of women facing a cancer treatment or another health disorder that may affect their reproductive potential.

In addition, 80% of the primary follicles presented normal morphology after vitrification in the metal cryovial, which is a better outcome, when compared with our own (Bos-Mikich *et al*., 2012) previous report using a metal caddy for vitrification following a similar methodology.

As discussed in that former report, primary follicles seem to be more sensitive to cryo-injury than the primordial structures.

Primordial follicles are smaller, the physical connections between the oocyte and the surrounding squamous cells are not yet established, which is a different situation from primary follicles, where cuboidal epithelial cells are attached to one another forming a well defined layer around the gamet (Bos-Mikich *et al*., 2012).

The pool of primordial and primary follicles represents the ovarian reserve of a woman and their integrity and developmental potential relate directly with the woman´s capacity to have her natural child.

Prepubertal girls and young women who face an immediate cancer treatment do not have many choices of fertility preservation, as embryo or oocyte banking.

Ovarian tissue cryobanking is becoming a feasible reality considering that more than a dozen of babies have been born after autotransplantation of cortical fragments (Donnez *et al*., 2004; Meirow *et al*.,2005; Andersen *et al*., 2008; von Wolff *et al*., 2009; Wallace *et al*., 2012). Thus, research concerning oncofertility should seek primarily to preserve the ovarian cortical layer, where the vast majority of primordial and primary follicles are nested to potentially guarantee these women future fertility and hormonal function.

When cryopreservation of ovarian tissue is considered, another important point to take into account is the stroma integrity.

The present study and our recent publication (Bos-Mikich *et al*., 2012) are in agreement with a previous report (Keros *et al*., 2009) showing that vitrification preserves stromal components without any major change from the fresh tissue morphology.

In our two systems using either an aluminium caddy or the steel cryovial, the stromal cells and extracellular matrix preserved their integrity, without gaps among collagen fibers and pycnotic-shrunken nuclei in the rewarmed tissues.

Despite the fact that these were not the main focus of our study, we also observed a certain proportion of growing antral follicles and their oocytes presenting a normal morphology after vitrification, which represents a positive finding seldom described in literature (Oktem *et al*., 2011). Growing antral follicles may be considered an additional source of oocytes, when induced to complete growth and maturation in an appropriate in vitro system, as it has been proven feasible using fresh ovarian tissue (McLaughlin *et al*., 2010).

Alternatively, oocytes lodged in antral follicles may be submitted to in vitro maturation procedures for subsequent fertilization and embryo transfer as performed in an IVM fertility treatment, as proved to be feasible using fresh ovarian tissue (Fasano *et al*., 2011). Since the first report of a successful vitrification protocol by Rall & Fahy (1985) several methodologies and carriers have been developed for the cryopreservation of oocytes, embryos and ovarian cortical tissue. However, most, if not all of them, use open systems that allow the direct contact of the specimen with LN2 or its vapour, a putative source of pathogens (Bielanski *et al*., 2000).

Additionally, traditional vitrification devices usually are small and delicate, factors that make it considerably fiddly to work and increase the risk of losing the biological material.

Another major point to take into consideration, when performing vitrification is the importance of cooling at the fastest possible rate to avoid intracellular ice creation and cytoskeleton disruption.

As mentioned in a recent review by Gosden (2011) “A physicochemical trade-off exists between cooling rates and solute concentrations needed for vitrification, which can be fine-tuned to reduce toxicity”. Considering that cooling rates vary according to the material employed as a vitrification device, different vitrification tools have been developed, open and closed systems that avoid direct contact with the LN2.

One closed system recently reported for ovarian tissue vitrification is a cryovial made of plastic (Sheikhi *et al*., 2011), a specimen which is not a good heat conductor as metals and the insulation slows the cooling rate. Having these facts in mind, we initially made a series of experiments (Bos-Mikich *et al*., 2012) using an aluminium foil caddy for vitrification, which proved to yield very good rates of primordial follicular survival. Those results prompted us to design a metal cryovial made of steel, which has the advantages of speedy cooling rate, tissues never enter in contact with LNi, it is strong and easy to handle.

These are real advantages when considering that some tissues may be stored for very long time, before the patient is physically capable of undergoing the transplant.

Tissues inside the cryovial have the additional advantage of being maintained vitrified for a more prolonged period than if they would have been vitrified in a plastic container, in case of an accidental exposure to RT.

A recent article also describes the use of a metal system for vitrification of caprine ovaries (Carvalho *et al*., 2013). However the authors report a considerably inferior follicular survival compared to our results, possibly because they vitrified hemi- or whole ovaries instead of small tissue fragments as we did.

The leak testing using a powdered dye represents an important validation assay of our closed system since it proved that the cryotube did not allow LN2 to enter inside the system and thus can avoid a putative contamination of the specimens during long-term storage in tissue cryobanks. In conclusion, we here show that clinical grade vitrification of ovarian cortex can successfully be performed in a closed metal container resulting in high survival rates of primordial and primary follicles.

The tissue never enters in direct contact with the LN2 and present results shall enable us to comply with future safety directives for tissue cryobanking in our country, as it is already a reality in Europe.

The vitrification methodology here described can safely be performed in any clinical setting, without the need of special apparatus and avoiding risks of contamination even for very long periods.
